# Is splenectomy an option for multiple relapses in a child with visceral leishmaniasis? A case report

**DOI:** 10.1590/0037-8682-0748-2020

**Published:** 2021-03-22

**Authors:** Marcos Adriano Garcia Campos, Andrey Salgado Moraes, Gustavo Ribeiro Féres Moraes Rêgo, Raphael Oliveira Lima Silva, Rebeca Aranha Barbosa Sousa, Yolande Pokam Tchuisseu, Gyl Eanes Barros Silva, Mônica Elinor Alves Gama

**Affiliations:** 1 Universidade Federal do Maranhão, Faculdade de Medicina, São Luís, MA, Brasil.; 2 Duke University, Duke Global Health Institute, Durham, North Carolina, United States.; 3 Universidade Federal do Maranhão, Hospital Universitário, São Luís, MA, Brasil.

**Keywords:** Splenectomy, Leishmaniasis, Recurrence

## Abstract

Visceral leishmaniasis (VL) is an infectious disease caused by *Leishmania* spp. The recurrence of the disease occurs, in general, in patients with decreased or loss of T-cell function, whether due to the use of corticosteroids, immunosuppressive disease, or another cause. In some cases, splenectomy may be a therapeutic option. However, the effectiveness of splenectomy is not well defined. This report describes the evolution of a pediatric patient with seven recurrences of VL, who relapsed post-surgery after drug therapy and splenectomy.

## INTRODUCTION

Visceral leishmaniasis (VL) is a highly fatal infectious disease caused by the intracellular parasite *Leishmania* spp*.* In the host, the parasite, which lives in macrophages, stays mainly in the liver, spleen, bone marrow, and lymph nodes. VL leads to splenomegaly, hepatomegaly, fever, pancytopenia, and susceptibility to infections and bleeding^1^.

VL is also prevalent in the pediatric population due to the immaturity of the immune response, lack of previous exposure, and a higher occurrence of risk factors such as malnutrition^2^. In Brazil, meglumine antimoniate (MA) and liposomal amphotericin B (LAmB) are currently the first-line drugs with a high cure rate of approximately 80%^3^. However, some patients have multiple recurrences or relapses, resulting from the failure of conventional treatment^1^.

The recurrence of the disease occurs, in general, in patients with decreased or loss of T-cell function, whether due to the use of corticosteroids, immunosuppressive disease or another cause. Recurrence rarely occurs in immunocompetent individuals and when it does, it is attributed to resistance to treatment or the improper delivery of the treatment^2^. In some cases, it is not possible to define the key factors that cause recurrence; hence, there is a need for additional research on individual characteristics associated with recurrence. 

In some cases, splenectomy may represent a therapeutic option; the drug levels in the spleen may not be high enough to eradicate the parasite and determine the clinical control of the disease. The removal of a large reticuloendothelial reserve has proved to be effective in reducing recurrence and the effects of hypersplenism, while improving hematological parameters^4^. However, the effectiveness of splenectomy has not been well defined^5^.

The purpose of this report is to describe the evolution of a pediatric patient with seven recurrences of VL, who relapsed post-surgery after drug therapy and splenectomy. 

## CASE REPORT

M. E. S. M., male, 6 years and 5 months old, from Itapecuru, state of Maranhão, Brazil, with a history of seven recurrences of VL.

The child was 3 years and 1 month old (March 2016) when he was first diagnosed with VL based on a clinical, epidemiological, and parasitological diagnosis, and he was treated at that time with MA for 30 days. Over the next two years, he presented with recurrences with clinical manifestations and laboratory alterations compatible with active disease. The myelogram was performed after three relapses. Amastigote forms of *Leishmania* spp. were found. He presented his first relapse in August 2016, and the MA treatment regimen was repeated for another 30 days. In December 2016, after the second relapse, the patient was treated with LAmB, with a total dose of 35 mg/kg for 10 days, without any change in the splenomegaly. The third recurrence in April 2017 was treated with amphotericin B deoxycholate (DAmB), at a total dose of 32 mg/kg. In August 2017, in the fourth relapse, LAmB was administered at a total dose of 50 mg/kg. After the fifth relapse, which occurred in March 2018, MA was used again for 30 days. After the sixth recurrence, which occurred on July 2018, a total dose of 48 mg/kg of LAmB was administered for 16 days. After each therapeutic regimen, he showed a regression of fever and an improvement in laboratory parameters, despite pancytopenia oscillations, while maintaining a hepatomegaly and a splenomegaly with little variations in their measurements (spleen persistently 10-12 cm from the left costal margin). 

An immunological evaluation was carried out at the Institute of Tropical Medicine in the state of São Paulo, Brazil. Flow cytometry and cytokine dosage revealed a specific immunosuppression for *Leishmania* spp., but with a satisfactory cellular and humoral immune response. No primary or secondary immunodeficiencies were documented. For six months, prophylaxis with MA was performed fortnightly, but without disease control. The seventh recurrence, which occurred in August 2018, was treated with LAmB, with a total dose of 50 mg/kg.

**FIGURE 1:**
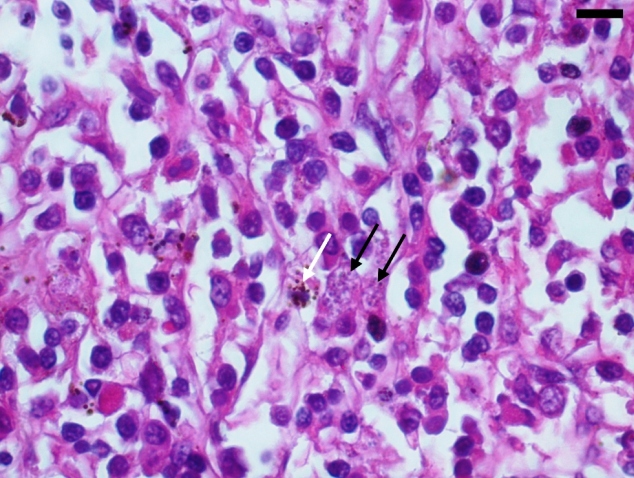
Amastigotes forms of the *Leishmania* parasite inside splenic histiocytes (black arrow), beside histiocytes with hemosiderin deposits (white arrow). Hematoxylin-eosin staining. Scale bar = 20 μm.

We opted to perform therapeutic splenectomy in August 2018, because of the persistence of splenomegaly, hepatomegaly, and pancytopenia. The spleen measured 20.5 × 12.0 × 8.0 cm and weighed 1.063 kg. Histopathology revealed the presence of amastigote forms of *Leishmania* spp. within the splenic histiocytes and perisplenic lymph nodes (Figure 1).

After the surgical procedure, the child showed clinical improvement. However, 10 months after splenectomy, the child had hepatomegaly, without pancytopenia and without fever. We performed a new myelogram that revealed *Leishmania* spp. amastigote forms with hypocellular marrow and preserved maturation. He was treated for 30 days commencing in March 2019 with Miltefosine, a drug supplied by the Pan American Health Organization and the Brazilian Ministry of Health upon request after being informed about the patient's condition. During this period, we performed an in-depth immunodeficiency assessment, which did not show lymphoproliferation. The prophylaxis with MA remained biweekly for six months (the patient was still receiving this medication at the time this report was being written, without any clinical manifestations or alterations in the laboratory parameters).

## DISCUSSION

Age is an important risk factor for VL for two main reasons: risk of developing the disease after infection and exposure to the *Lutzomyia longipalpis* vector. The risk of unsatisfactory response to treatment is 5 to 10 times higher in children, the main victims of this disease in epidemic periods; therefore, special attention should be given to treatment in children. Additional research is important to understand the conditions that determine changes in the immune response, especially since children are known to have immature immune systems^6^.

The treatment failure rate for MA in immunocompetent VL patients is low, therefore MA remains the drug of choice for cases without risk or severity factors. However, some patients require a higher dose than that recommended to achieve a cure, and the criteria for declaring a failure in response after administration of 40 doses of MA may need to be modified in some situations. LAmB, on the other hand, has the potential for toxicity and its use should be restricted. In cases of relapse, a total dose of up to 50 mg/kg of LAmB may be necessary, as in the case described above. However, given the risks associated with LAmB and the lack of effectiveness of MA in some situations, it is likely that the oral drug miltefosine, which emerged as one of the most recently adopted therapeutic options, can alter the recommendations for the treatment of VL^6^.

Much still remains to be known about the factors involved in the immune response of this disease, but it is known that the balance of the parasite-host relationship would be the cause of both the elimination and the pathogenic process of leishmaniasis; after specific treatment, the parasite that remains viable in macrophage phagosomes is controlled by the formation of the granuloma, determining the clinical and non-parasitological cure by restoring the production of Th1 type cytokines^7^. Thus, in cases of recurrence, it is necessary to assess the association of VL with some type of acquired or primary immunodeficiency, specific or not, as observed in this case report.

The effect of splenectomy in patients with VL has not been well defined. In two immunocompetent adult cases with multiple leishmaniasis relapses and exhausted drug therapy, splenectomy was performed as surgical treatment with a curative purpose and showed a good clinical response to hypersplenism and other clinical repercussions^8^. Other cases in which splenectomy had been successful in minimizing the effects of hypersplenism and curing refractory cases of VL have been described in India^5^ ^,^ ^9^.

However, two cases were reported in which parasites remained present in the lymph nodes after clinical cure^10^, demonstrating that the reticuloendothelial system, especially the spleen, can be a reservoir of infected cells, thus justifying surgical intervention in specific cases^8^. The success of splenectomy was observed in a report by Dutra et al. and was associated with the removal of large amounts of parasites from the reticuloendothelial system and the correction of hypersplenism, a fact that was not observed in the patient in this case, since there were clinical recurrences and changes in the laboratory parameters, even after splenectomy. In this context, the possibility of an immunodeficiency profile was raised after results from the low cytometry and cytokine measurement showed evidence of specific immunosuppression for *Leishmania* spp.^11^.

There is a consensus among experts that antileishmania therapy should advance in a drug combination regimen to prevent acquired resistance. However, there are no recommendations for specific combination regimens yet^12^.

The case was studied using the national health references, described in clinical protocols and therapeutic guidelines for VL; however, it was a challenge given that it was an unusual case with multiple recurrences of VL. Splenectomy, despite being a therapeutic resource for cases with VL, has not been effective in controlling the disease, a finding that suggested the presence of an individual condition causing or influencing the ineffectiveness of the immune response to control the multiplication of the parasite. Despite the limited effectiveness of various treatment options presented in this case report, miltefosine, though not yet included in the protocol of the National Ministry of Health, stands out both in this case study and in international studies as a major therapeutic advance for VL, positioning itself as a promising therapeutic strategy.

## References

[1] Dos-Santos WLC, Paglari C, Santos LG, Almeida VA, Silva TLV, Coutinho JJ (2014). A case of conventional treatment failure in visceral leishmaniasis: leukocyte distribution and cytokine expression in splenic compartments. BMC Infect Dis.

[2] Lagadinou M, Dimitropoulou D, Assimakopoulos S, Davoulos G, Marangos M (2013). Recurrent visceral leishmaniasis in an immunocompetent patient: a case report. J Med Case Reports.

[3] Romero GAS, Costa DL, Costa CHN, Almeida RP, Melo EV, Carvalho FG (2017). Efficacy and safety of available treatments for visceral leishmaniasis in Brazil: A multicenter, randomized, open label trial. PLoS Negl Trop Dis.

[4] Alon D, Chowers M (2012). Successful therapeutic splenectomy in an HIV patient with relapsing visceral leishmaniasis. Int J STD AIDS.

[5] Mukhopadhyay B, Sarkar AK, Dasgupta A, Mukhopadhyay M, Chowdhury MM, Sarkar S (1993). Drug - resistant childhood visceral leishmaniasis. Is splenectomy a solution?. Pediatr Surg Int.

[6] Santos MA, Marques RC, Farias CA, Vasconcelos DM, Stewart JM, Costa DL (2002). Predictors of an unsatisfactory response to pentavalent antimony in the treatment of American visceral leishmaniasis. Rev Soc Bras Med Trop.

[7] Bacellar O, Carvalho E (2005). Imunopatogênese da Leishmaniose Visceral. Gaz Méd Bahia.

[8] Reinaldo LGC, Araújo RJC, Diniz TM, Moura RD, Costa DL, Eulálio KD (2020). Recurrent kala-azar: report of two cured cases after total splenectomy. Rev Inst Med Trop S Paulo.

[9] Lyngdoh E, Jain SC, Barua P (1971). Splenectomy in treatment of drug-resistant Kalazar. J Indian Med Assoc.

[10] Dereure J, Duong TH, Lavabre-Bertrand T, Cartron T, Bastides F, Richard-Lenoble D (2003). Visceral leishmaniasis. Persistence of parasites in lymph nodes after clinical cure. J Infect.

[11] Dutra RA, Dutra LF, Reis MO, Lambert RC (2012). Splenectomy in a patient with treatment-resistant visceral leishmaniasis: a case report. Rev Soc Bras Med Trop.

[12] den Boer ML, Alvar J, Davidson RN, Ritmeijer K, Balasegaram M (2009). Developments in the treatment of visceral leishmaniasis. Expert Opin Emerg Drugs.

